# The coastal environment and human health: microbial indicators, pathogens, sentinels and reservoirs

**DOI:** 10.1186/1476-069X-7-S2-S3

**Published:** 2008-11-07

**Authors:** Jill R Stewart, Rebecca J Gast, Roger S Fujioka, Helena M Solo-Gabriele, J Scott Meschke, Linda A Amaral-Zettler, Erika del Castillo, Martin F Polz, Tracy K Collier, Mark S Strom, Christopher D Sinigalliano, Peter DR Moeller, A Fredrick Holland

**Affiliations:** 1 Hollings Marine Laboratory, NOAA National Ocean Service, Charleston, SC 29412, USA; 2 Woods Hole Oceanographic Institution, Woods Hole Center for Oceans and Human Health, Woods Hole, MA 02543, USA; 3 Water Resources Research Center, University of Hawaii, Honolulu, HI 96822, USA; 4 Rosenstiel School for Marine and Atmospheric Sciences, University of Miami, Miami, Florida 33149, USA; 5 Department of Environmental and Occupational Health Sciences, University of Washington, Seattle, WA 98105-6099, USA; 6 The Josephine Bay Paul Center for Comparative Molecular Biology and Evolution, Marine Biological Laboratory, Woods Hole Center for Oceans and Human Health, Woods Hole, MA 02543, USA; 7 Civil and Environmental Engineering, MIT, Woods Hole Center for Oceans and Human Health, Cambridge, MA 02139, USA; 8 Northwest Fisheries Science Center, NOAA Fisheries, Seattle, WA 98112, USA; 9 Atlantic Oceanographic and Meteorological Laboratory, NOAA Office of Oceanic and Atmospheric Research, Miami, FL 33149, USA; 10 Cooperative Institute of Marine and Atmospheric Studies, University of Miami, Miami, FL 33149, USA

## Abstract

Innovative research relating oceans and human health is advancing our understanding of disease-causing organisms in coastal ecosystems. Novel techniques are elucidating the loading, transport and fate of pathogens in coastal ecosystems, and identifying sources of contamination. This research is facilitating improved risk assessments for seafood consumers and those who use the oceans for recreation. A number of challenges still remain and define future directions of research and public policy. Sample processing and molecular detection techniques need to be advanced to allow rapid and specific identification of microbes of public health concern from complex environmental samples. Water quality standards need to be updated to more accurately reflect health risks and to provide managers with improved tools for decision-making. Greater discrimination of virulent versus harmless microbes is needed to identify environmental reservoirs of pathogens and factors leading to human infections. Investigations must include examination of microbial community dynamics that may be important from a human health perspective. Further research is needed to evaluate the ecology of non-enteric water-transmitted diseases. Sentinels should also be established and monitored, providing early warning of dangers to ecosystem health. Taken together, this effort will provide more reliable information about public health risks associated with beaches and seafood consumption, and how human activities can affect their exposure to disease-causing organisms from the oceans.

## Introduction

Bodies of water, particularly the coastal oceans and the Great Lakes, provide a source of food, employment, recreation and residence, and are the first defense from various natural and man-made hazards and disasters. Maintaining these as functional and healthy ecosystems is essential for our future well-being. Currently 50% of the world population lives in towns and cities within 100 km of the coast [1]. These coastal areas are impacted through pollution inputs due to changes in land use and hydrology, with vast amounts of our wastes entering on a daily basis. Ocean and estuarine ecosystems can therefore impact the extent to which humans are exposed to microbial pathogens, which include both marine-indigenous pathogens and externally introduced microbial contaminants. These pathogens can be found in association with marine animals, phytoplankton, zooplankton, sediments and detritus. Environmental factors, including salinity, temperature, nutrients and light, influence the survival and sometimes the proliferation of pathogens.

**Figure 1:**
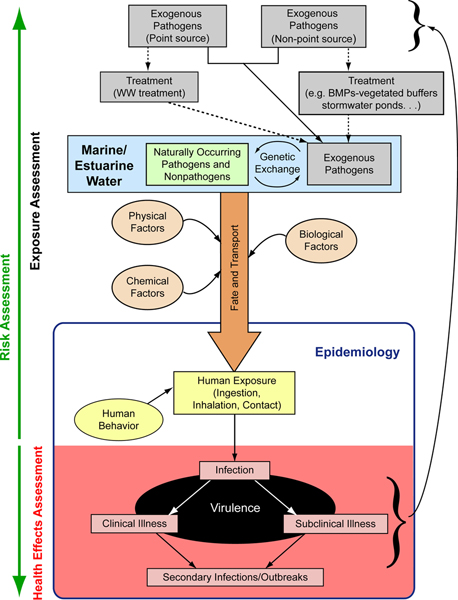
**Relationships between pathogens, the environment and human health**. WW treatment = wastewater treatment; BMPs = best management practices.

Recent research relating oceans and human health is addressing a range of issues in environmental health microbiology (Figure 1), including examinations of the sources and sinks of pathogens, human exposures, effects of development and management practices, and the expression of disease. New detection methods have been developed and tested, which represent not only a comparison of different approaches but take into account idiosyncrasies of different geographical areas (e.g. tropical vs. temperate regions), as well as the standardization of sample collection and processing methods. This work has broadened our perspectives on the types of microbial pathogens present in the ocean and the importance of non-point sources of contamination in the environment. In this manuscript we present several of the current challenges to understanding the impacts of microbes of public health concern in the coastal environment, including (1) indicator organisms, and their relationship to water quality, (2) non-point sources of contamination, (3) direct pathogen detection, (4) virulence, (5) non-enteric diseases resulting from recreational water use, (6) animals and environments as sentinels of water quality, and (7) zoonotic and emerging diseases.

## Discussion

### Water quality and indicators of fecal contamination

The Clean Water Act (CWA), as amended by the Beaches Environmental Assessment and Coastal Health (BEACH) Act of 2000, requires coastal and Great Lakes states and territories to adopt bacterial water standards as protective as EPA's 1986 bacterial standards [2]. These standards are based on concentrations of fecal indicator bacteria (FIB) such as *E. coli *or enterococci. However, monitoring for these indicators is not always effective for determining when streams and coastal waters are contaminated with sewage because FIB can take up residence in the environment and may even multiply under certain conditions. Another problem with current water quality standards is the known difference between fate and transport characteristics of FIB compared to pathogens. Indicator bacteria are more sensitive to inactivation through treatment processes and have also been shown to be more sensitive to inactivation by sunlight than viral and protozoan pathogens. Sunlight inactivation varies seasonally, daily and at different geographic and climatic regions [3]. As a result, the concentrations of FIB in water samples measured after disinfection or measured in environmental waters are not reliable assurances that human pathogens in water have been reduced to levels that will not cause infections in swimmers [4].

Additional issues regarding the use of indicators occur with regard to the sample substrate and the sources of contamination. For over three decades, beach sands and sediments in tropical and subtropical environments have been documented to contain high concentrations of bacterial indicators. Studies conducted in Hawaii and Guam [5-7] and in Puerto Rico [8] have shown that in the absence of any known sources of human/animal waste, enterococci and *E. coli *are consistently present and recovered in high concentrations. In South Florida, river bank soils and beach sands have been implicated as the source of indicator microbes to the water column [9-11], and experimentation has documented that soil moisture mediated by tidal cycles is a key factor in facilitating regrowth [12]. Turnover due to current may also play an important role. Subsurface sands and sediments may serve as a refuge and a source when sands are reworked by wave action or erosion.

The significance of beach sands and other environmental sources is not necessarily limited to the sub-tropics. For example, sands and sediments have been implicated as a bacterial source at marine beaches in California [13-16] and at freshwater beaches of Lake Michigan [17], Lake Huron [18] and Lake Superior [19]. Again, soil moisture has been implicated in persistence and possible regrowth of indicator microbes [20]. More recently, aquatic plants including *Cladophora *[21] and epilithic periphyton communities [22] have also been identified as potential contributors to fecal indicator persistence and regrowth in temperate regions.

**Figure 2:**
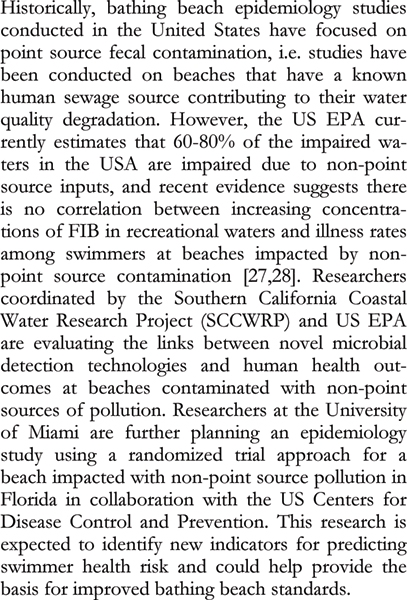
**Bathing beach epidemiology studies**. References [27,28].

Alternative indicators are being proposed and evaluated to better identify risks to human health and to improve monitoring strategies. Ideally, these alternative indicators cannot multiply under environmental conditions, are present in low concentrations in unimpacted environmental samples, and are present in high concentrations in sewage. Proposed alternatives include bifidobacteria [23], *Clostridium perfringens *[24], human viruses [25] and F-RNA coliphages [26]. The validation of new water quality indicators requires the correlation of the presence and abundance of the organism with human disease, often gastroenteritis. These correlations are made through epidemiology studies where study subjects (beach goers) report on disease symptoms and severity, and measurements of the organisms in the environment are conducted (Figure 2). It is expected that these techniques for identifying fecal contamination will allow for improved risk assessments and more informed monitoring.

Alternative indicators are also being proposed to help track sources of fecal pollution. Microbial source tracking methods are commonly classified as library-dependent or library-independent. A library is a database of characteristics (e.g. genetic fingerprints, antibiotic resistance profiles) of microbes from known sources. In library-dependent analyses, characteristics of isolates from contaminated waters are compared to the library to find matches, thereby identifying the source of contamination. Library-independent approaches entail analysis of water samples for source-specific markers (e.g. human-specific bacterial strains) to help identify sources. These approaches do not require building and maintenance of representative databases for each study area. Appropriate study design and application of microbial source tracking tools can be complicated, particularly for library-based methods [29,30]. However, source tracking technologies are rapidly advancing and can already provide useful insight for managers trying to mitigate contamination.

### Non-point sources of contamination

Non-point sources of contamination are of significant concern with respect to the transport of pathogens and their indicators into the marine environment. Point source pollution enters the environment at a distinct location through a direct route that is often easily identified, while non-point source pollution is generally diffuse and intermittent in nature, and occurs through a non-direct route. Examples include runoff from urban and agricultural areas, leaking septic systems and sewerage lines, combined sewer overflows, discharges from boats and atmospheric deposition of aerosols. We currently have a limited understanding of the actual pathogen loading of the coastal environment due to non-point source contamination, nor do we fully understand the consequences of this loading. Addressing non-point source contamination is one of the main challenges for future research. Further assessment of the effect of non-point sources on the loading of pathogens in the marine environment needs to include not only pathogen abundance, but an indication of the potential risk to human health and the ability to track the source of contamination.

**Figure 3:**
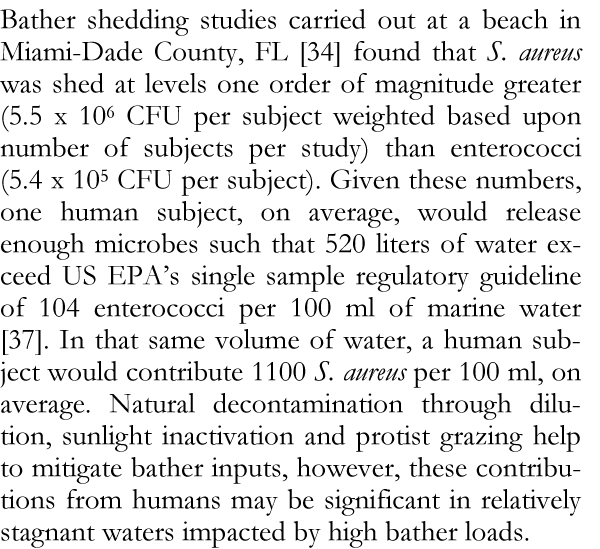
**Marine bather shedding study**. References [34,37].

Swimmers may themselves be sources of microbes in water. Bather shedding studies conducted in freshwater have long shown that humans release significant numbers of microbes, in particular enterococci and *Staphylococcus aureus *[31-33]. Elmir *et al. *[34] conducted the first experimental human bathing study in marine waters, and results were consistent with those found in freshwater (Figure 3). As *S. aureus *is isolated from human sewage in relatively low numbers (10^3 ^CFU/100 ml) relative to enterococci (10^4 ^to 10^5 ^CFU/100 ml) [35], it can be used as an indicator to predict human bather impacts, which would include the combined effects of bather density, mixing, and dilution. The use of *S. aureus *as a potential supplemental indicator is especially significant as studies have shown an association between illness in swimmers and bather density [30,36].

### Direct pathogen detection

**Figure 4:**
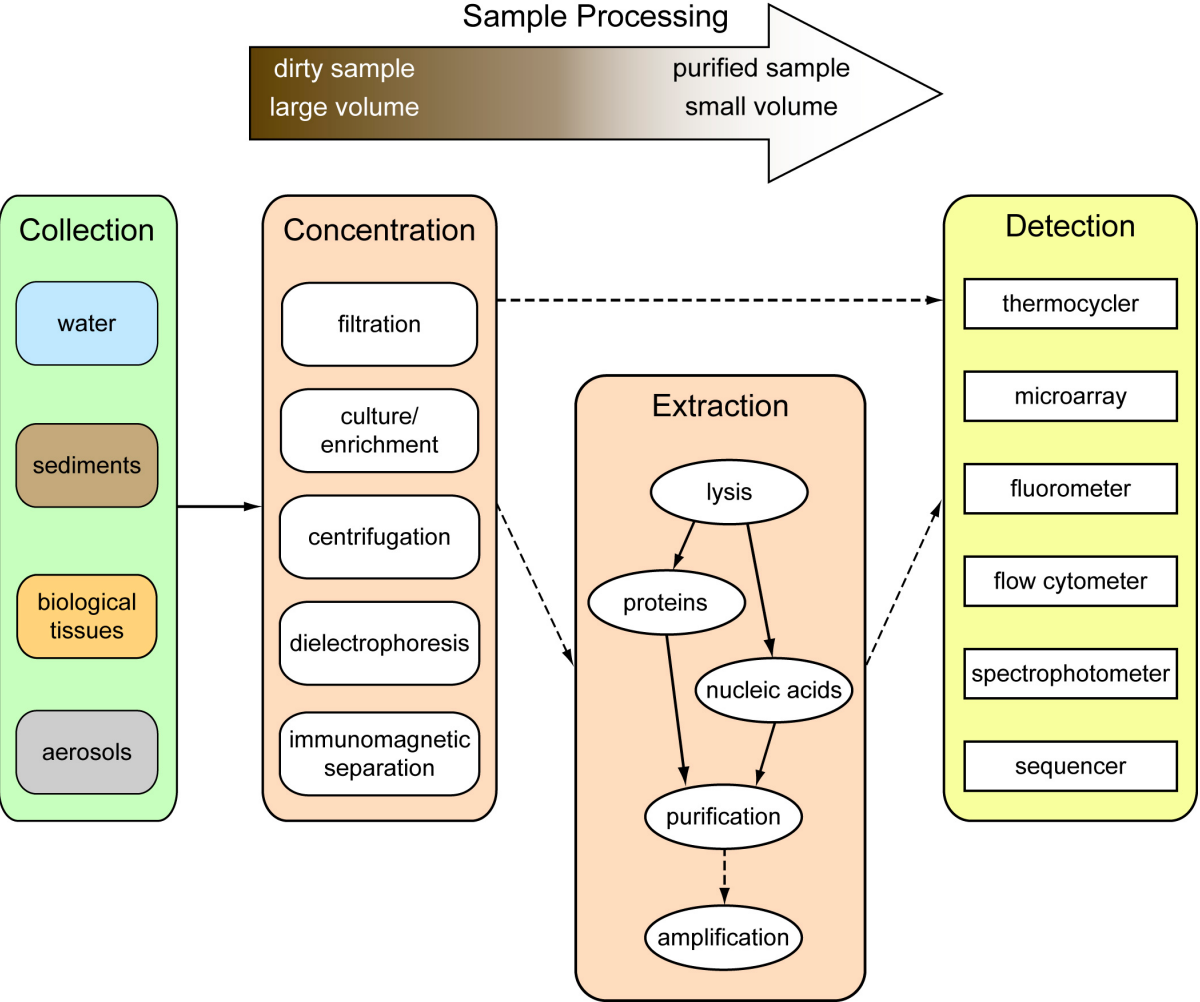
**Common approaches for molecular detection of pathogens from environmental samples**. It is important to use appropriate controls and to quantify recovery efficiencies of each step depicted.

**Figure 5:**
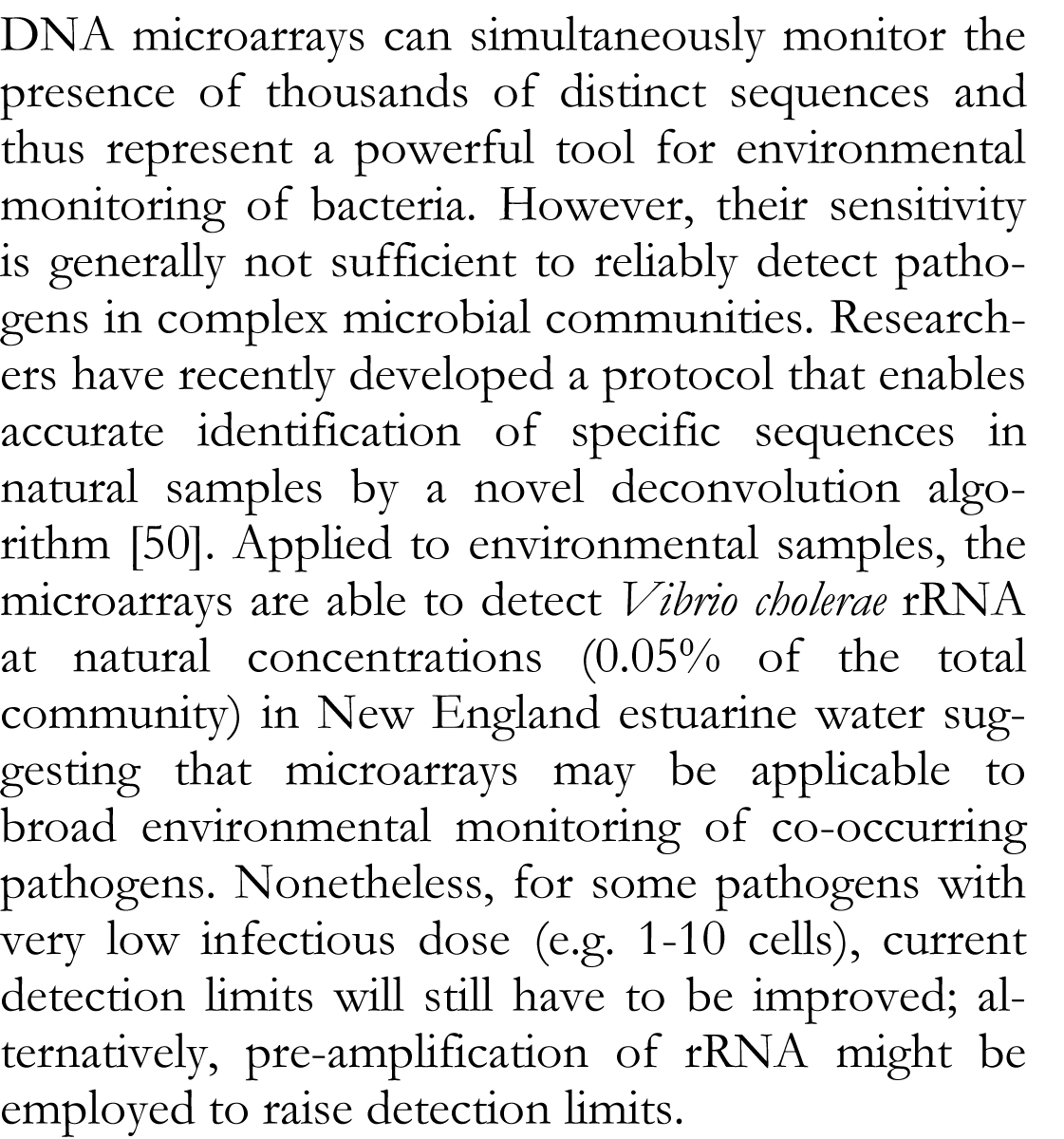
**Pushing the limits of DNA microarrays**. Reference [50].

Given that presence and prevalence of indicators does not often correlate well with risk of infection from environmental pathogens, direct monitoring for pathogens is often suggested. Detection of pathogens from environmental samples is gaining momentum through the development of molecular technologies (Figure 4). Approaches are typically based upon measurement of specific sequences of nucleic acids (DNA or RNA), or through structural recognition of pathogens or biomarkers [38]. While the detection of pathogen DNA is not necessarily indicative of viability or even infectivity, it does represent a rapid and specific method. Probably the most popular methodology involves using the polymerase chain reaction (PCR) to amplify nucleic acids to detectable levels. This technology is allowing researchers to rapidly and specifically target microbes of public health concern, including those that were previously unexamined because of our inability to culture them. New molecular assays have been introduced for detection of FIB [39,40], bacterial pathogens [41,42], viral pathogens [25,43], and protozoan parasites [44,45]. Additionally, recent improvements in detection technologies are allowing simultaneous detection of multiple targets in a single assay [[46-49]; Figure 5]. As with new indicators, nucleic acid based detection of specific pathogens will need to go through testing to determine what level of detection is associated with unacceptable human health risk.

Despite the advances in detection technologies, sample collection and processing remains an issue for direct pathogen detection [51,52]. Improved filtration methods [53], electrochemical methods [49], immunochemical methods [54-56] and nanotechnologies [57,58] may enable better cell sorting and improve microbial isolation techniques, but each of these has its own challenges to overcome. Low recovery efficiencies associated with concentrating cells and extracting nucleic acids continue to decrease overall sensitivity for molecular targets. Isolation of microbes from complex samples is also problematic. Soil and sediment samples show high levels of heterogeneity and vary significantly with regard to physical and chemical composition, making the establishment of standardized protocols problematic. These variables represent challenges not only for quantitative recovery of nucleic acids, but for enzymatic manipulation of the resulting sample. Regardless, it is imperative that researchers calculate recovery efficiencies for their concentration and nucleic acid extraction protocols, and for amplification or other detection steps. Further, inhibition of PCR or other reactions should be quantified with an internal control, and concentration estimates should be appropriately adjusted.

It remains to be determined how direct pathogen detection and more rapid technologies will alter monitoring strategies. Given the advances made in measurements during the last two decades, monitoring programs will likely include measurements for a suite of microbes. A tiered monitoring strategy may also be a viable option [14]. In a tiered approach, the first step could involve using the simplest and most practical tests for contamination, perhaps utilizing a rapid test for fecal indicators. Tier two may involve adding methods to differentiate human from animal sources of pollution, and the tier three test, if necessary, would measure for specific pathogens. Supplemental microbial measurements could also be added, including indicators of disease transmitted via other routes of infection, including respiratory routes (e.g. adenovirus, *Legionella*), direct human shedding (e.g. *S. aureus*), and oral ingestion or wound infections from indigenous microbes (e.g. *Vibrio*). This tiered approach would not necessarily be appropriate for characterization of transient events, unless samples are appropriately archived for each level of analysis.

### Understanding virulence

Methodologies that simply identify the presence of a pathogenic species in many cases may not improve existing tools since not all strains of infectious bacteria are equally pathogenic. Strain variation can arise through several mechanisms, including mutation, genomic rearrangements, inter and intragenic recombination, or acquisition of genes from mobile genetic elements including transducing bacteriophages. Maintenance of these genetic changes occurs if they result in a selective advantage for survival in a given environmental niche [59,60]. Therefore, while a significant proportion of the population may not be virulent, there is always the potential for emergence of more infectious strains. This pattern of virulence is especially true of many marine bacteria including members of the *Vibrio *spp. For example, the majority of environmental *V. cholerae *strains are non-toxigenic and do not cause disease unless the cholera toxin gene is acquired via phage transduction [61]. Strains of the shellfish-borne pathogen *V. vulnificus *can now be genetically differentiated into one group more likely to cause clinical infections and another group that is less infectious and forms the majority of environmental strains [62-65]. Similarly, while *V. parahaemolyticus *is ubiquitous and often found in shellfish during harvest periods, relatively few genotypically distinct strains are responsible for most outbreaks of human infections [66]. There also appears to be an increase or emergence of more virulent *V. parahaemolyticus*, with increasing numbers of human infections arising from ingestion of raw oysters containing relatively few bacteria, although increased exposure of susceptible populations may play a part as well. Regardless, rapid detection tools must not only be sensitive enough to identify low numbers of a pathogen, but also be discriminatory enough to detect the presence of specific strains of a pathogen capable of expressing virulence genes required for human infection. The application of comparative genomics with contemporary molecular pathogenesis and virulence studies will aid in the identification of genes that will serve as sensitive and accurate markers of risk [67].

**Figure 6:**
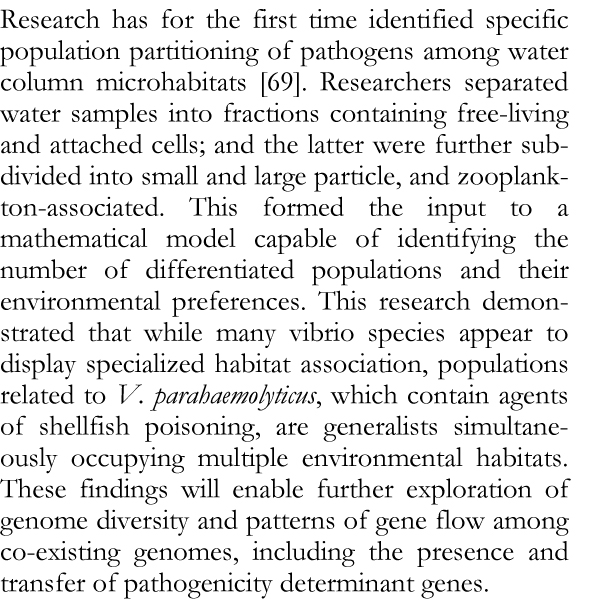
**Population-level partitioning of vibrios among (micro) habitats in the water column**. Reference [69].

Genomics is also assisting population level studies to estimate the extent to which genotypes associate with defined environmental microhabitats and the probability of genetic exchange (of virulence factors and other genes) among co-existing microbes [68]. Particularly important is the identification of environmental reservoirs of virulent strains (Figure 6). Indeed, pathogenicity towards humans likely arises among indigenous marine bacteria due to adaptation to a specific marine microhabitat rather than selection for infection of the human body. This is because there is insufficient feedback from infected humans to environmental populations of the pathogen (with perhaps the notable exception of cholera in epidemic areas) to act as positive selection on virulence genes. For example, a specific *V. cholerae *surface protein contributes to the ability of the bacterium to attach to the chitin exoskeleton of zooplankton [70], an important environmental survival mechanism. While the same protein has also been shown to aid attachment to the human gut mucosa [70], it can be argued that the primary selective driving force for increased attachment is microhabitat adaptation. Overall, the ability to evade predation or establish zoonotic associations may predestine a microbe's ability to infect humans, although this is currently poorly understood [38].

In addition to virulence factors, it is also important to understand how bio-signaling molecules and other chemical compounds affect pathogenicity. The marine microbial community is responsible for the synthesis of highly bioactive secondary metabolites. These compounds are used to communicate (quorum sensing), defend (toxicity), or otherwise provide a competitive advantage to a select community [71-73]. In some cases these compounds augment the chemical behavior of other chemical or biological processes. This is often observed in cases of biofilm formation and/or incorporation of metals into metabolites [74,75] where enhanced pathogenicity or antibiotic resistance has been noted [76,77]. For example, when a biofilm created by a given microbial community is removed, often times old generation antibiotics become effective. Previously they simply could not cross the film. Investigating the interaction of these chemicals is critical in understanding the role the microbial community plays in human health related issues.

### Non-enteric diseases from water or aerosol transmission

There have been several epidemiology studies regarding the potential transmission of non-gastrointestinal illnesses associated with recreational use of water. While an increased risk of gastroenteritis for swimmers is often found, several non-enteric infections have also been associated with exposure, including respiratory illness, and ear, eye and skin infections [78-83]. In one study, 63.7% of the 683 participants reporting illnesses indicated respiratory symptoms [79]. In most of these studies, the risk of non-enteric infections in swimmers increased with either exposure to urban runoff or declining water quality due to pollution or sewage.

Some pathogenic microorganisms are naturally present in freshwater environments (*Aeromonas hydrophila*, *Naegleria fowleri*, *Legionella pneumophila*), while other human pathogenic species are indigenous to marine and brackish waters (*V. cholerae*, *V. vulnificus*, *V. parahaemolyticus*, *V. alginolyticus*). Pathogens naturally present in water may be transmitted to humans via inhalation, contact or ingestion of water or contaminated food. Examples of sources of non-enteric pathogens in environmental waters include animal urine for *Leptospira *spp. and shedding from human skin for *S. aureus*. In addition, Khan et al. [84] have discovered *Pseudomonas aeruginosa *in open ocean environments. *P. aerugionosa *is an opportunistic pathogen that has been regarded to be widely present in terrestrial and freshwater environments. This report further illustrates the potential significance of the ocean as a reservoir for pathogenic isolates of traditionally non-indigenous bacteria.

**Figure 7:**
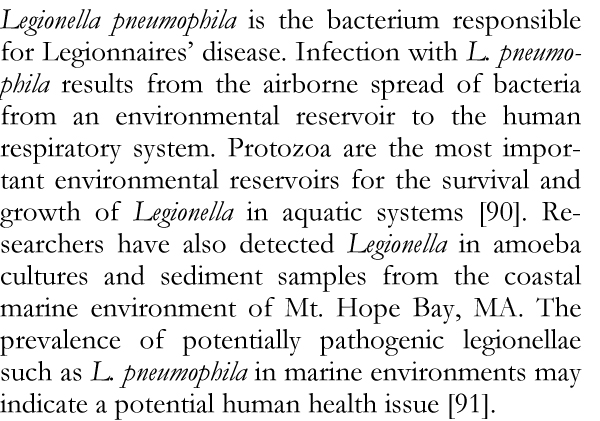
***Legionella pneumophila *in the marine environment**. References [99,91].

Another potential source of non-enteric pathogens are free-living amoebae. Free living amoebae are natural reservoirs of many types of bacteria such as *Legionella *spp., *Burkholderia pickettii*, *Vibrio cholerae*, *Myobacterium avium *and *Listeria monocytogenes *[85,86]. It is known that *Acanthamoeba *species are natural aquatic reservoirs of several intracellular pathogens such as *Legionella*, *Chlamydia *and *Mycobacterium *[87,88]. Laboratory studies with *Acanthamoeba castellanii *have shown the ability of *Francisella tularensis*, the agent of tularemia, to survive in amoebal cysts; however, the potential for its survival under various adverse conditions in these cysts needs to be examined [89]. Studies have also shown that naturally occurring marine amoebae can harbor some of these pathogens (Figure 7), but their prevalence and the extent to which they harbor other pathogens is unknown. Amoebae in cooling towers and water treatment facility biofilms are considered the primary reservoir for pathogenic legionellae, not only providing refuge for the bacteria but also enhancing the infectivity of the microbe [92,93]. The level of human health risk these associations represent from the marine environment is unknown, and part of this problem is potentially the lack of correlation between marine exposure and the onset of symptoms several days to a week later.

Studies on the ecology and distribution of non-enteric pathogens in the marine environment are necessary to understand their potential threat to human health. Yet one of the biggest challenges remains effective monitoring. When the source of the etiological agents for non-enteric diseases is not fecal in origin, traditional monitoring for FIB will unlikely be successful in predicting the presence or absence of these pathogens. Although for many non-enteric pathogens there are methods for their specific detection and quantification from natural samples, there are no standards relevant for predicting the level of risk for human infection. Thus, research should be targeted towards establishing these standards for non-enteric pathogens where appropriate, or else efforts should be made toward public education about infections where human behavior plays a key role.

### Sentinel species and sentinel habitats

A variety of marine species and habitats are excellent indicators or *sentinels *of environmental stress and potential health threats for humans. They provide information about how events in the environment may affect humans and animals. Sentinel species and habitats fall into at least three categories: (1) wildlife or habitats that tend to "sample" or concentrate contaminants, toxins, and/or pathogens from their environment and thus may provide more biologically relevant indicators of possible effects than water sampling alone; (2) wildlife with diets and/or physiologies at least partially similar to those of humans and which therefore may demonstrate early indications of potential health effects of environmental levels of contaminants, toxins and pathogens before they show up in humans (a biological early warning system); and (3) habitats that encompass key ecosystem components and are subjected to early and often high pollution exposure, thereby indicating potential effects at systems or community levels and on people.

#### Biologically relevant sentinels in the environment

Once pathogens enter the marine environment, they can be further concentrated by the action of filter feeding organisms such as mussels, clams and oysters. Mussels and oysters, in particular, are implicated more than any other marine animal in seafood illnesses. Since they are sessile filter feeders inhabiting the benthic environment, they bioaccumulate both metal and organic contaminants [94,95], as well as concentrate microbial organisms including human pathogens [96-99]. The evaluation of the presence and distribution of pathogens among marine bivalves is critical to determining the present and future risk to human health by better understanding the nature of the interaction between pathogens and shellfish.

#### Potential effects of environmental contamination

Marine organisms, particularly marine mammals, can also be sensitive sentinel species to warn of impending human health problems from ocean-borne pathogens. Causes of mass mortality events in marine mammals have included viruses, bacteria and protozoa [100]. Monitoring for either emerging or recurring health problems in marine animals may provide information that can be used as a measure of ocean health that could also indicate the potential for future human health issues. The ability to use marine mammals as sentinels for pathogens important to ecosystem and human health requires appropriate tools and protocols to accurately test for and track those pathogens in sentinel populations and the ecosystem. In addition to being useful for detecting zoonotic diseases that can affect human health, marine mammals are also shown to be useful sentinel species for assessing health risks from natural toxins (i.e. algal toxins) and persistent chemical contaminants (e.g. polybrominated diphenyl ethers (PBDEs) and other organochlorines).

#### Habitats

**Figure 8:**
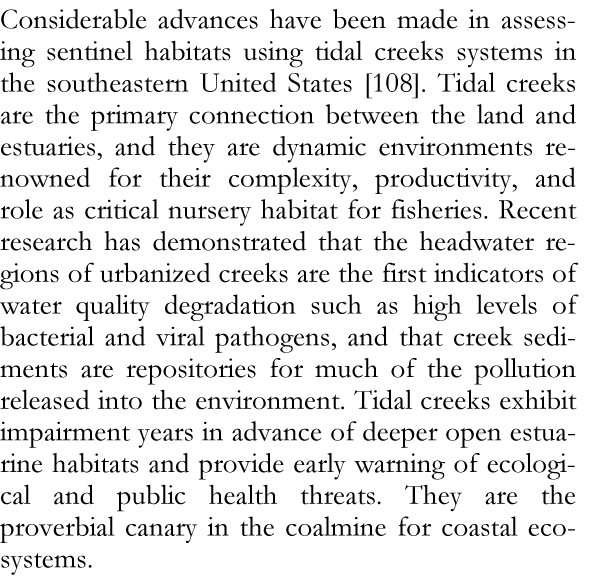
**Tidal creeks as sentinel habitats**. Reference [108].

Habitats serve as first repositories and impact zones for terrestrial runoff and thereby serve as sentinels of land-use impacts on adjacent coastal environments and human communities. One of the earliest symptoms of broad scale coastal ecosystem impairment has historically been declines in the amount and condition of critical habitats that are sensitive to changes in environmental conditions. Notable examples include sea grass beds, oyster reefs, kelp forests, coral reefs, and tidal creeks, including the estuarine wetlands associated with and draining into them. These sentinel habitats or first responders generally decline in extent and condition from years to decades before system-wide impairment is documented by routine environmental quality monitoring activities [101-103]. Unfortunately, the scientific knowledge to understand the warning signals provided by sentinel habitats has only recently become available [[104-107]; Figure 8].

While use of sentinel species and habitats provide many advantages over routine water and sediment sampling, they also have some important limitations. For example, migratory species such as some marine mammals, may integrate environmental conditions over a large coastal or ocean area, but may not provide as reliable information about specific localities. On the other hand, sedentary species such as oysters and specific local habitats like tidal creeks may give a very accurate picture of localized effects of contaminants, toxins and pathogens, but gaining a picture of more widespread impacts may require sampling of sentinels in numerous locations. Nonetheless, sentinels allow us to sample at several trophic levels and to develop understanding of how contaminants, toxins and pathogens may be passed, accumulated and/or biomagnified through food chains that include humans. Because they integrate the broad range of environmental conditions to which they are exposed, sentinel species and habitats can give a much better picture of the cumulative health effects of degraded coastal ecosystems than any other measure. They also provide unique opportunities to study how disease-causing materials may be passed directly from organism to organism and from mother to offspring, and the kinds of impacts associated with such passage, including cellular and gene-level responses. Such studies are generally much more difficult to conduct in humans.

### Zoonotic and emerging diseases

**Figure 9:**
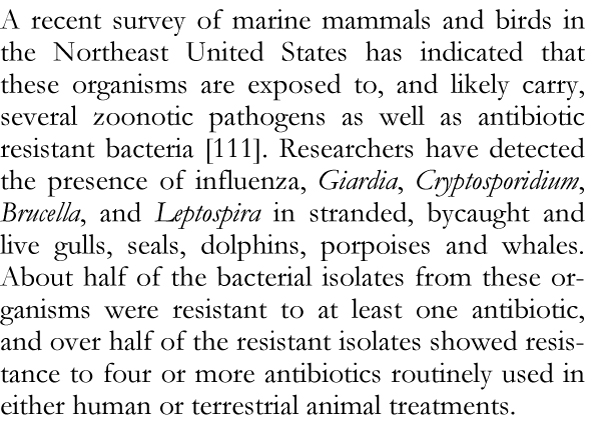
**Marine animal zoonoses**. Reference [111].

An estimated 75% of emerging infectious diseases are zoonotic [109], and anthropogenic influence on ecosystems appears to be a common factor in the emergence and reemergence of zoonotic pathogens [110]. In the marine environment proper, bacterial, viral, fungal and protozoal pathogens that can infect humans have been detected in a range of marine animals, including pinnipeds, dolphins, cetaceans and otters (Figure 9). Bacteria such as *Brucella*, *Leptospira *and *Mycobacterium *have been shown to infect humans handling marine mammals [112,113], while others such as *Clostridium*, *Burkholderia *(formerly *Pseudomonas*), *Salmonella *and *Staphylococcus *have the potential to be transmitted to humans. Calicivirus and influenza A have been documented to occur in pinnipeds [114,115], and *Blastomyces *have been detected in dolphins [116]. California sea lions and ringed seals have been found to harbor *G. lamblia *and *Cryptosporidium *spp. [117,118] and *Giardia *cysts have been found in fecal material from harp seals (*Phoca groenlandica*), grey seals (*Halichoerus grypus*), and harbour seals (*Phoca vitulina*) from the St. Lawrence estuary in eastern Canada [119,120]. Pinnipeds have also been shown to harbor *Salmonella *and *Campylobacter *species, including strains that are resistant to multiple antibiotics [121,122]. Shorebirds are also potentially able to transmit parasites to humans. Canada geese carry several enteric human pathogens including *G. lamblia*, *Camplyobacter jejuni *and *C. parvum *[123,124]. Therefore, it seems possible that shorebirds feeding in an area that is contaminated by a sewer outfall may be yet another source of concentrated pathogen input to either shellfishing areas or recreational areas.

Analysis of the interaction between host animals (domesticated or wildlife) and the coastal watershed, the natural reservoirs in marine habitats, and the survival, prevalence and proliferation of the pathogens are a rational area of concern for disease emergence. New methods for direct or indirect detection of microorganisms are contributing to the detection of zoonoses [125,126], but there is still a lack of understanding regarding the public health significance.

## Conclusion

Oceans directly and indirectly impact the extent to which humans are exposed to disease-causing organisms. Recent research has greatly enhanced our understanding of the relationships between pathogens, coastal and marine environments, and human health. Assays for rapid detection of water-borne and shellfish-borne pathogens have been developed and optimized, and progress toward simultaneous and real-time detection is underway. Genetic factors associated with virulence are being discovered so that lethal strains can be specifically identified. Similarly, genetic targets are being revealed that help differentiate human from nonhuman sources of fecal contamination. Alternative indicators are being proposed to more accurately assess risks to human health, and new measures of contamination are being linked to health outcomes to help improve management criteria. In some instances, marine organisms and coastal habitats act as reservoirs for newly-introduced terrestrial pathogens and can contribute to disease transmission. These organisms and habitats are acting as sentinels for health status within a given ecosystem and can also help warn of threats from emerging pathogens. Overall, this research is leading to a greater understanding of how oceans affect human health, and how humans themselves influence this process.

## List of abbreviations used

US EPA: United States Environmental Protection Agency; FIB: fecal indicator bacteria.

## Competing interests

The authors declare that they have no competing interests.

## Authors' contributions

This manuscript was originally conceived during a breakout session at an Oceans and Human Health Initiative Center Directors Meeting held in Woods Hole, MA in April 2007. All of the listed authors then contributed text, and Rebecca Gast and Jill Stewart served to compile and edit the resulting document. All authors read and approved the final manuscript.
